# Multilocus Sequence Typing Reveals New Insights into the Population Structure and Genetic Diversity of *Lactococcus* spp. from Brazilian Fish

**DOI:** 10.3390/microorganisms14051131

**Published:** 2026-05-16

**Authors:** Guilherme Campos Tavares, Sarah Portes Carneiro, Angelo Carlo Chaparro Barbanti, Angélica Emanuely Costa do Rosário, Helena Caldeira Matos, Cynthia Rafaela Monteiro da Silva Maia, Henrique Lopes Costa, Renata Catão Egger, Luiz Fagner Ferreira Nogueira, Júlio César Câmara Rosa, Felipe Luiz Pereira, Fabiana Pilarski, Silvia Umeda Gallani, Esteban Soto, Carlos Augusto Gomes Leal, Henrique César Pereira Figueiredo

**Affiliations:** 1 Department of Preventive Veterinary Medicine, School of Veterinary Medicine, Federal University of Minas Gerais (UFMG), Belo Horizonte 31270-901, Minas Gerais, Brazil; sarahportes7@gmail.com (S.P.C.); caldeiramatosh@gmail.com (H.C.M.); henriquelopes.costa17@gmail.com (H.L.C.); egger.rc@gmail.com (R.C.E.); fagnerfnogueira@outlook.com (L.F.F.N.); jcbhrama@gmail.com (J.C.C.R.); leal.cag@gmail.com (C.A.G.L.); figueiredoh@yahoo.com (H.C.P.F.); 2 Postgraduate Program in Aquaculture, Nilton Lins University, Manaus 69058-030, Amazonas, Brazil; angelocarloch@gmail.com (A.C.C.B.); angelicamanu0807@gmail.com (A.E.C.d.R.); cynthiarafaeladasilva.2023@gmail.com (C.R.M.d.S.M.); silviaugallani@gmail.com (S.U.G.); 3 Department of Quantitative Health Science, Mayo Clinic, Jacksonville, FL 32224, USA; pereira.felipe@mayo.edu; 4 Department of Neuroscience, Mayo Clinic, Jacksonville, FL 32224, USA; 5 Laboratory of Microbiology and Parasitology of Aquatic Organisms, Aquaculture Center of São Paulo State University (UNESP), Jaboticabal 14884-900, São Paulo, Brazil; fabiana.pilarski@unesp.br; 6 Department of Medicine & Epidemiology, School of Veterinary Medicine, University of California, Davis, CA 95616, USA; sotomartinez@ucdavis.edu

**Keywords:** *Lactococcus*, native fish species, ornamental fish, sequence type, clonal complex, phylogenetic relationships

## Abstract

Lactococcosis has emerged as an economically and ecologically significant disease in aquatic animals worldwide. This study employed multilocus sequence typing (MLST) to investigate the genetic diversity of *Lactococcus* spp. strains from Brazilian fish species and evaluate their phylogenetic relationships with global isolates to elucidate potential epidemiological connections involving multiple host species and distinct geographic regions. A total of 55 isolates from different laboratories had their DNA extracted, followed by the amplification and sequencing of the internal fragments of seven housekeeping genes (*als*, *atpA*, *tuf*, *gapC*, *gyrB*, *rpoC* and *galP*). Sequence types (STs) and clonal complexes (CCs) were defined. An unrooted neighbor-joining phylogenetic tree was generated using allele profiles from this study and those previously reported from other aquatic animal species. The isolates comprised 29 STs (11 previously reported, 18 novel ones), which were grouped into species-specific CCs: CC5 (*L. formosensis*); CC4, CC17, CC62 (*L. garvieae*); CC24, CC29, CC97 (*L. petauri*). Considerable genetic divergence was observed, with *L. formosensis* and *L. garvieae* forming heterogeneous populations, while *L. petauri* was more homogeneous. These findings describe the MLST structure of the sampled isolates and should be interpreted as hypothesis-generating rather than population-level estimates of genotype prevalence. Phylogenetics confirmed groupings within the CCs and revealed additional phylogenetic clustering patterns. In conclusion, the Brazilian *Lactococcus* spp. strains analyzed in this study constitute a genetically diverse population based on their STs. MLST and phylogenetic analysis demonstrated genetic relatedness between the *L. garvieae* and *L. formosensis* isolates from this study and those from other aquatic animal species. In contrast, all the STs identified for *L. petauri* in this study were unrelated to the MLST lineages responsible for outbreaks in Brazilian Nile tilapia (*Oreochromis niloticus*) and North American rainbow trout (*Oncorhynchus mykiss*). This suggests that piscine *L. petauri* populations in the Americas evolved from distinct ancestral origins.

## 1. Introduction

Lactococcosis has emerged as an economically and ecologically significant disease in aquatic animals worldwide [1]. Disease outbreaks and associated mortality have been linked to infections caused by *Lactococcus formosensis*, *L. garvieae* and *L. petauri* in various fishes and prawn species, particularly in aquaculture systems [2,3,4,5,6,7,8]. Among the lactococcosis-causing bacteria (LCBs), *L. petauri* has been responsible for the most significant economic losses in commercial rainbow trout (*Oncorhynchus mykiss*) [9] and Nile tilapia (*Oreochromis niloticus*) [3] production in the Americas. Nevertheless, LCBs have been detected in a wide range of fish species, some of which are susceptible to natural infection or experimental challenge [10,11,12,13,14,15,16,17,18,19,20,21]. In addition to aquatic animals, these three pathogens have also been identified in terrestrial animals including humans, products destined for human consumption, and in the environment [22,23,24,25,26].

Given the broad range of hosts and wide geographic distribution of these pathogens, genetic characterization studies have become a critical tool in epidemiological investigations. Such studies help elucidate the pathogen’s genetic structure and assess genetic relatedness or the divergence among isolates [23]. Different molecular typing methods have been employed for the genotyping of strains of LCBs. Among the sequencing-based methods, MLST is the most widely adopted for assessing genetic diversity in bacterial pathogens, including those that affect aquatic host species [27,28].

MLST is a molecular typing technique that relies on sequencing internal fragments of housekeeping genes and has been extensively used to determine phylogenetic relationships among bacterial isolates [23], infer ancestral genotypes and trace evolutionary lineages [22]. For LCBs, the seven housekeeping genes analyzed—along with their corresponding proteins—are *als* (α-acetolactate synthase), *atpA* (ATP synthase α subunit), *tuf* (elongation factor EF-Tu), *gapC* (glyceraldehyde-3-phosphate dehydrogenase), *gyrB* (DNA gyrase β subunit), *rpoC* (RNA polymerase β subunit) and *galP* (galactose permease) [22]. The combination of alleles from these genes defines an allelic profile, which corresponds to a sequence type (ST). Genetic relatedness among isolates can be inferred by comparing these allelic profiles. Allele and ST designations can be used to classify strains into clonal complexes (CCs) or lineages, thus providing insights into population structure and evolutionary dynamics [29]. Furthermore, curated MLST databases, particularly those hosted by PubMLST [30], offer standardized nomenclature and facilitate phylogenetic analysis to infer evolutionary relationships [29]. This approach enables the differentiation of LCB strains isolated from a variety of hosts and from different geographic regions [3,22,23,24,25,26,31,32]. Based on previously published MLST data, the predominant STs identified in fish include: ST10 (cobia, rainbow trout), ST13 (rainbow trout), ST14 (rainbow trout), ST15 (trout), ST16 (yellowtail), ST17 (yellowtail), ST24 (Nile tilapia), ST34 (red tilapia), ST39 (tilapia), ST41 (bighead carp), ST43 (yellowtail), ST46 (Nile tilapia), ST47 (Nile tilapia), ST95 (greater amberjack, European seabass, gilthead seabream), ST139 (European seabass), ST157 (European seabass), and ST158 (European seabass) [3,23,26,31,32,33].

In Brazil, fish farming has become the leading economic activity within the aquaculture sector, with Nile tilapia serving as the primary commodity. Additionally, the production of native (particularly from the orders Characiformes and Siluriformes) and exotic fish species (such as carps, rainbow trout and striped catfish) has expanded nationwide [34]. However, the intensification of farming systems has facilitated the emergence of infectious diseases, especially those of bacterial origin. Among these, *L. petauri* has emerged as a major pathogen causing production losses in tilapia farming [3]. Furthermore, this and other *Lactococcus* species have been increasingly detected in native fish across the country [21]. Despite the importance of lactococcosis in national fish production, information on the genetic diversity of Brazilian strains remains scarce. A recent study evaluated the genetic diversity of isolates from LCBs derived from native fish species using PCR-based DNA fingerprinting techniques (REP-, RAPD-, and BOX-PCR) [21]. The result is consistent with significant genetic heterogeneity among *L. garvieae* strains, whereas *L. petauri* isolates exhibited a more homogeneous population [21]. To date, MLST analysis in Brazil has been restricted to *L. garvieae* and *L. petauri* isolates obtained from Nile tilapia from different commercial farms, revealing that there are only three STs (ST24, ST46 and ST47) in circulation [3]. However, no MLST data are available for LCBs strains from other fish species in the country, raising the question of whether these strains could belong to the same STs. This represents a critical knowledge gap since it remains unclear whether the genetic structure of *Lactococcus* spp. infecting non-Nile tilapia hosts mirrors that reported in tilapia-associated strains, or whether the same genotypes are shared between fish farms and wild fish populations. Furthermore, MLST-based surveillance is ideal for assessing potential cross-species transmission, elucidating potential epidemiological connections among diverse host species and across geographic regions, and supporting evidence-based biosecurity measures in Brazilian aquaculture.

Therefore, this study aimed to evaluate the genetic diversity and population structure of Brazilian LCB strains isolated from multiple fish species using the MLST approach, and to compare these findings with data from other aquatic host strains available in the PubMLST database.

## 2. Materials and Methods

### 2.1. Bacterial Strains and Identification

This study used a total of 55 *Lactococcus* spp. strains, comprising *L. formosensis* (n = 7), *L. garvieae* (n = 20) and *L. petauri* (n = 28) isolates. The strains were obtained from 16 fish species (*Arapaima gigas*, *Brycon amazonicus*, *Carassius auratus*, *Cichla* sp., *Colossoma macropomum*, *Hoplias macrophtalmus*, *Hoplias malabaricus*, *Lophiosilurus alexandri*, *Pangasianodon hypophthalmus*, *Phractocephalus hemioliopterus*, *Pseudoplatystoma corruscans*, *Pseudoplatystoma fasciatum* and a hybrid of *Pseudoplatystoma*, *Pterophyllum scalare*, *Trichogaster lalius*, *Xiphophorus maculatus*) from wild populations and commercial farms in six Brazilian states (Amazonas, Bahia, Mato Grosso do Sul, Minas Gerais, Pará and São Paulo) between 2012 and 2024 (Table 1) [21,35,36,37,38,39]. The isolates were obtained during routine diagnostic investigations of bacterial infections in fish conducted by the Laboratory of Aquatic Animal Diseases (AQUAVET, Veterinary School, Federal University of Minas Gerais, Belo Horizonte, Brazil), Laboratory of Applied Microbiology of Aquatic Organisms (LAMAO, Nilton Lins University, Manaus, Brazil), Laboratory of Microbiology and Parasitology of Aquatic Organisms (LAPOA, Aquaculture Center of São Paulo State University, São Paulo, Brazil), and Fisheries Institute (IP, São Paulo, Brazil). These isolates comprise the entire set of *Lactococcus* spp. strains obtained from the aforementioned laboratories and maintained in a culture collection, excluding those originating from Nile tilapia, representing a total of 26 different sampled sites. Furthermore, all the selected isolates were previously identified to the species level using matrix-assisted laser desorption ionization time-of-flight (MALDI-TOF) mass spectrometry (Bruker Daltonics, Bremen, Germany) [37] with the Bruker MALDI Biotyper database (v13.0.0.2) followed by *gyrB* sequencing [22] at the Laboratory of Aquatic Animal Diseases. The isolates were stored at −80 °C in BHI broth with 15% glycerol until use.

### 2.2. DNA Extraction

The selected *Lactococcus* spp. strains were cultured from cryopreserved stocks on de Man, Rogosa and Sharp (MRS) agar (Merck, Darmstadt, Germany) and incubated at 28 °C for 72 h under aerobic conditions. Colonies were harvested and resuspended in 180 µL of lysis buffer (20 mg/mL lysozyme, 20 mM Tris-HCl [pH 8.0], 2mM EDTA, and 1.2% Triton X-100), followed by incubation at 37 °C overnight. Bacterial DNA was extracted using the Maxwell^®^ 16 Tissue DNA Purification kit (Promega, Madison, WI, USA) in accordance with the manufacturer’s protocol. The DNA concentration of each isolate was measured in duplicate using a NanoDrop^®^ 2000 spectrophotometer (Thermo Fisher Scientific, Wilmington, DE, USA). The elution buffer from the extraction kit was used as the blank. The 260/280 nm absorbance ratio was used to assess sample purity, with values ranging from 1.8 to 2.0 deemed acceptable for subsequent steps. DNA samples were stored at −20 °C until further analysis.

### 2.3. Multilocus Sequence Typing

For the MLST analysis, the isolates were characterized by sequencing internal fragments of seven housekeeping genes, following a modified version of the previously described protocol [22]. In summary, PCR amplification was performed using the Gotaq^®^ PCR Core System kit (Promega, Madison, WI, USA) in 25 µL reaction volumes containing 100 ng of DNA template (2 µL) and 23 µL of PCR mix (1× PCR buffer, 0.2 µM of each primer [Table 2], 0.2 mM dNTPs, 1.5 mM MgCl_2_, 0.625 U of DNA polymerase, and nuclease-free water). The primers were synthesized and purified by Invitrogen (Thermo Fisher Scientific, Wilmington, DE, USA). The strain LG01-13 and nuclease-free water were used as positive and negative controls, respectively, in all of the assays.

Amplification of *als*, *tuf*, *gapC*, *gyrB*, *rpoC* and *galP* was conducted in a 96-well thermal cycler (Veriti^®^, Applied Biosystems, Foster City, CA, USA) under the following conditions: initial denaturation at 95 °C for 5 min; 35 cycles of 94 °C for 45 s, 56–58 °C (primer-specific, see Table 2) for 45 s, and 72 °C for 70 s; and a final extension at 72 °C for 5 min. The *atpA* gene was amplified using a touchdown protocol: initial denaturation at 95 °C for 5 min; 3 cycles of 95 °C for 60 s, 56 °C for 135 s, and 72 °C for 75 s; followed by 30 cycles of 95 °C for 35 s, 56 °C for 75 s, and 72 °C for 75 s; with a final extension at 72 °C for 7 min.

Amplicons were size verified by capillary electrophoresis (QIAxcel Advanced System, Qiagen, Hilden, Germany) using the QX DNA Screening kit (Qiagen) according to the manufacturer’s protocol. PCR products were then purified using the Agencourt AMPure^®^ XP kit (Beckman Coulter, Brea, CA, USA). Sanger sequencing was performed using the BigDye^®^ Terminator v3.1. Cycle Sequencing kit (Applied Biosystems) in a genetic analyzer (ABI 3500, Applied Biosystems). Sequences were checked by visual inspection of electropherograms and quality scores using Geneious Prime v.2022.2.2 (Dotmatics, Boston, MA, USA). Subsequently, contigs were assembled and manually curated using the same software.

### 2.4. Data Analysis

To determine the allelic profiles and STs for each isolate, the assembled contigs were analyzed against the *L. garvieae* typing scheme in the PubMLST database (https://pubmlst.org/organisms/lactococcus-garvieae accessed on 5 August 2025) [30]. The numbers of alleles (haplotypes), polymorphic sites, haplotypic diversity, and nucleotide diversity were calculated using the software DnaSP v.6.12 [40]. Additional *Lactococcus* spp. strains isolated from aquatic animals with publicly available genome sequences in GenBank databases [41,42], but without prior ST designations in the literature, were selected for analysis (Table S1). The corresponding FASTA sequences were retrieved and subsequently uploaded to the PubMLST database via the BIGSdb platform for automated in silico analysis. The combination of the seven allele numbers for each isolate was used to define ST. Novel allelic profiles and STs were assigned to both the newly sequenced strains in this study and the previously deposited genomes.

The genetic relationships among the LCB isolates were inferred using the goeBURST algorithm [43,44], performed in PHYLOViZ software v.2.0 [45]. Clonal complexes were defined as closely related STs based on single-locus variants (SLVs) using the PHYLOViZ software’s default parameters. The novel STs and CCs identified in this study were designated with the prefix ‘n’ preceding the ST, or the CC number.

To examine the phylogenetic relationships among the LCB isolates, we constructed an unrooted phylogenetic tree incorporating both novel allele profiles from this study and previously reported alleles from diverse animal aquatic species worldwide (Table S2). The seven housekeeping gene sequences were concatenated and the isolate sequences composed by all the loci were aligned using ClustalW implemented in MEGA12 v.12.1 [46]. Phylogenetics relationships were inferred using the Neighbor-Joining method [47] based on the Tamura–Nei model [48]. Branch support was assessed using 1000 bootstrap replicates to evaluate topological robustness [49]. Evolutionary analyses were conducted in MEGA12 [46]. The resulting phylogenetic trees were visualized using iTOL v.6 online tool [50].

ST and CC distributions were summarized as counts and proportions according to bacterial species, host species, Brazilian state/region, production origin, tissue and year. ST-level diversity was quantified using Simpson’s diversity index discriminatory index [51], calculated as D = 1 − [Σnj(nj − 1)]/[N(N − 1)], where nj is the number of isolates belonging to the ST and N is the total number of isolates. Approximate 95% confidence intervals were calculated for the diversity estimates. Exploratory associations between STs/CCs and metadata were assessed only for interpretable contingency tables. Fisher’s exact test was used for 2 × 2 comparisons, and permutation chi-square tests were used for multi-category tables. A nominal significance threshold of α = 0.05 was adopted; when multiple exploratory tests were considered jointly, Benjamini–Hochberg false-discovery-rate adjustment was applied. Missing metadata were not imputed. Isolates lacking complete MLST profiles were excluded from analyses requiring ST or CC assignment.

## 3. Results

### 3.1. MLST Analysis

**Figure 1:**
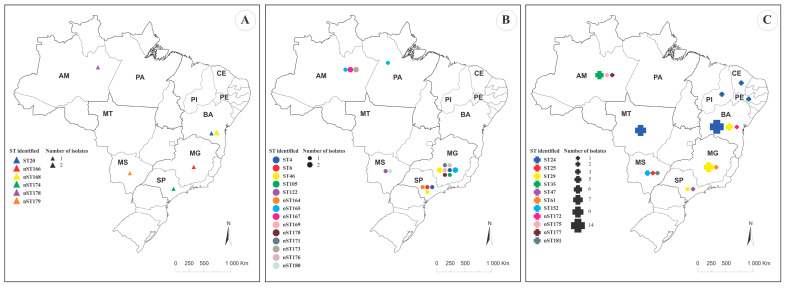
Map of the distribution of *L. formosensis* (**A**), *L. garvieae* (**B**) and *L. petauri* (**C**) sequence types (ST) identified in this study according to Brazilian state. Different colors represent the different STs and symbol sizes are proportional to the number of isolates per ST. *Lactococcus garvieae* ST46, *L. petauri* ST24 and ST47 Nile tilapia-derived isolates were added to demonstrate Brazilian genetic diversity.

Evaluation of the DNA polymorphism for each allele within the MLST scheme revealed high haplotype diversity paired with low nucleotide diversity (Table 2). This pattern suggests a rapid population expansion from a small number of individuals alongside the generation of new mutations, supporting the hypothesis of varying evolutionary rates across the evaluated loci. Consequently, our MLST analysis revealed that the allelic profiles of the 55 LCB strains evaluated in this study grouped the isolates into 29 distinct STs (Table 3). The map of the distribution of *L. formosensis*, *L. garvieae*, and *L. petauri* STs is shown in Figure 1. Overall, the 55 isolates were assigned to 29 STs, of which 18/29 (62.1%) were novel. The overall ST-level Simpson diversity index was 0.929 (95% CI, 0.883–0.975). ST diversity (permutation test, *p* < 0.001) and CC diversity (permutation test, *p* < 0.001) differed among species.

Analysis of the 67 LCB genome sequences isolated from aquatic animals and subjected to MLST analysis in PubMLST identified 20 STs, including 10 novel STs. Only the ERR5094895 strain (from rainbow trout in Poland) lacked an assigned ST due to the absence of the *als* gene in its genome sequence (Table S1).

**Figure 2:**
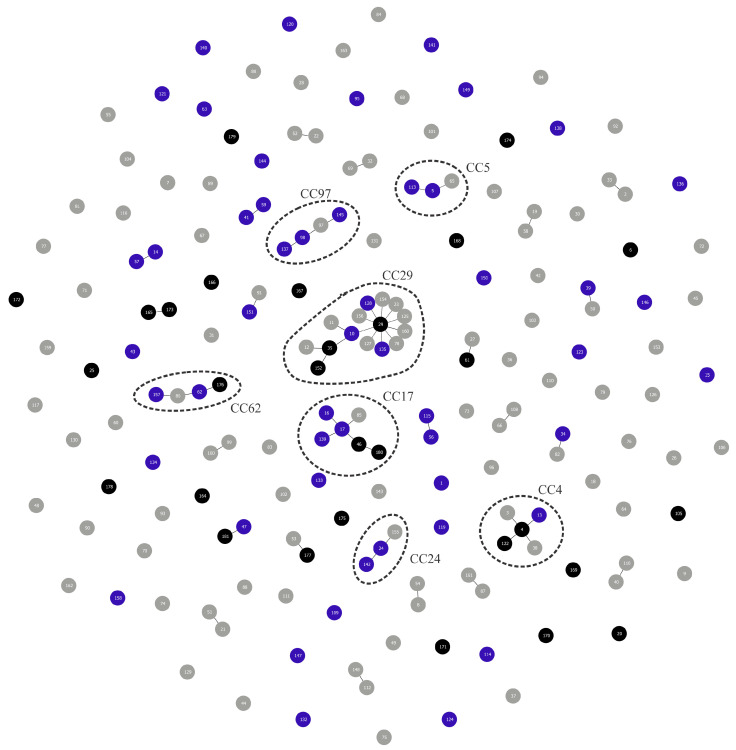
Global optimal eBURST analysis of all sequence types (ST) available to date in the *Lactococcus garvieae* typing scheme in the PubMLST database. Each circle represents an ST. Each number inside a circle represents an ST. Blue circles represent ST isolated from fish or prawns, black circles represent ST observed in this study, and grey circles represent other STs deposited in the PubMLST database. Black lines represent single-locus variants. STs highlighted in dashed lines form a clonal complex.

The *L. formosensis* strains used in this study were grouped into 6 different STs, including one previously reported (ST20) and five novel STs (nST166, nST168, nST174, nST178 and nST179). All of these STs were characterized as singletons (Table 3, Figure 2). The Simpson’s diversity index (SDI) value was 0.952 (95% CI, 0.857–1.000).

The *L. garvieae* strains grouped into 14 different STs, including five previously reported STs (ST4, n = 2; ST6, n = 1; ST46, n = 1; ST105, n = 1; ST122, n = 1) and nine novel STs (nST164, n = 1; nST165, n = 4; nST167, n = 2; nST169, n = 1; nST170, n = 1; nST171, n = 1; nST173, n = 2; nST176, n = 1; and nST180, n = 1). ST4 and ST122 were grouped into CC4 (3/55 isolates). ST46 and nST180 clustered into CC17 (2/55 isolates), and the nST176 belongs to CC62 (1/55 isolates). The nST165 and nST173 clustered together, but differed only in the *galP* gene allele (a 4-nucleotide divergence), without forming a distinct clonal complex. Finally, ST6, ST105, nST164, nST167, nST169, nST170 and nST171 were characterized as singletons (Table 3, Figure 2). The SDI value was 0.953 (95% CI, 0.903–1.000). For *L. garvieae*, exploratory permutation testing did not provide robust evidence of an association between ST and geographic region (*p* = 0.070), although a non-significant trend was observed. Likewise, there is no significant association between region and CC (*p* = 0.156).

The *L. petauri* strains grouped into nine different STs, including five previously reported STs (ST25, n = 1; ST29, n = 13; ST35, n = 6; ST61, n = 1; ST152, n = 3) and four novel STs (nST172, n = 1; nST175, n = 1; nST177, n = 1; nST181, n = 1). ST29, ST35 and ST152 were grouped into CC29 (22/55 isolates). In this isolate collection, ST35 was detected only among isolates from Amazonas, whereas ST152 was detected only among isolates from Mato Grosso do Sul. These patterns should be interpreted as descriptive spatial clustering within the sampled isolates, rather than evidence of state-specific STs. ST29, however, was detected in different hosts and across various geographical regions. ST61, nST177 and nST181 clustered with ST27, ST53 and ST47, respectively, but did not form distinct CCs. Finally, ST25, nST172 and nST175 were characterized as singletons (Table 3, Figure 2). The SDI value was 0.746 (95% CI 0.612–0.880). In *L. petauri*, exploratory permutation testing indicated that ST distribution differed by geographic region (*p* < 0.001) and state (*p* = 0.003). This pattern was mainly driven by ST35 among Amazonas isolates, ST152 among Mato Grosso do Sul isolates, and ST29 among isolates from Bahia, Minas Gerais and São Paulo. Because the contingency table was sparse, this result was interpreted as descriptive clustering within the analyzed collection rather than evidence of population-level geographic specificity. However, when considering CC level, there is no clear evidence of region (*p* = 0.879) or state (*p* = 0.918) distribution.

Overall, the collection showed high ST-level diversity, but diversity differed among species. *L. formosensis* and *L. garvieae* showed high ST richness relative to sample size, whereas *L. petauri* showed lower ST-level diversity because most isolates belonged to ST29 and nCC29. Table 4 summarizes the ST diversity and CC information for all studied isolates, as well as stratified by species. The observed clonal complexes (CCs) were significantly associated with specific species: CC5 with *L. formosensis*; CC4, CC17, and CC62 with *L. garvieae*; and CC24, CC29, and CC97 with *L. petauri* (*p* < 0.001).

### 3.2. Phylogenetic Relatedness Between Fish Isolates

**Figure 3:**
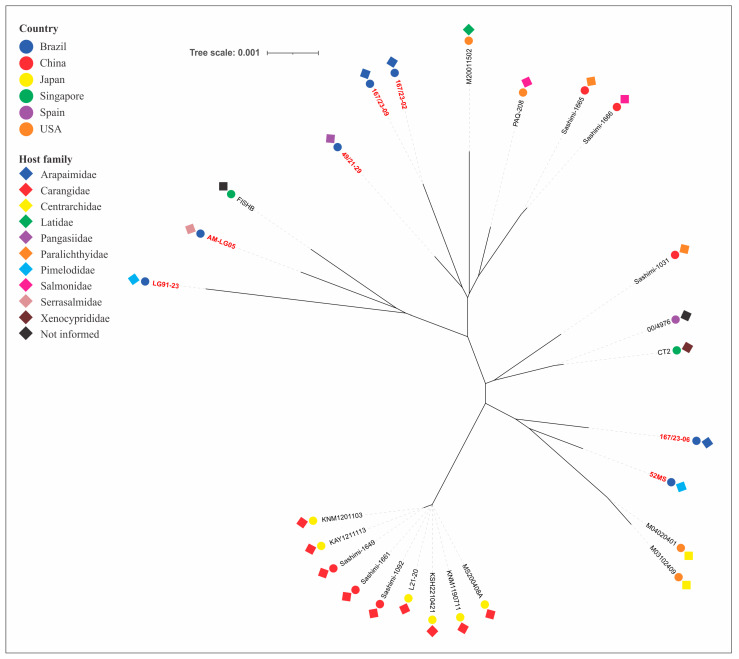
Phylogenetic tree of *Lactococcus formosensis* strains obtained from aquatic animals. The isolate’s name in red denotes strains from this study. The colors of the circles and diamonds indicate the isolate’s country of origin and host origin, respectively.

**Figure 4:**
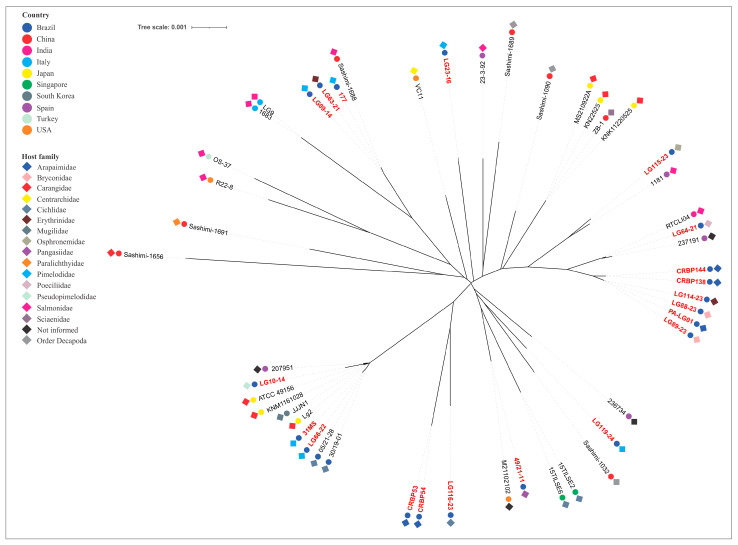
Phylogenetic tree of *Lactococcus garvieae* strains obtained from aquatic animals. The isolate’s name in red denotes strains from this study. The colors of the circles and diamonds indicate the isolate’s country of origin and host origin, respectively.

**Figure 5:**
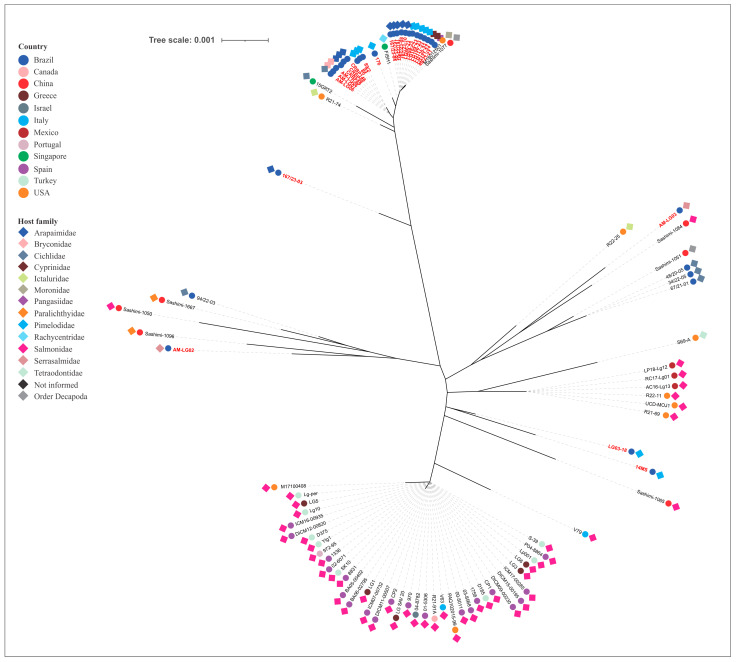
Phylogenetic tree of *Lactococcus petauri* strains obtained from aquatic animals. The isolate’s name in red denotes strains from this study. The colors of the circles and diamonds indicate the isolate’s country of origin and host origin, respectively.

The phylogenetic tree, constructed from concatenated MLST allele sequences of piscine *L. formosensis*, *L. garvieae*, and *L. petauri*, are presented in Figure 3, Figure 4 and Figure 5, respectively.

The *L. formosensis* strains clustered into five major groups, with the strains reported in this study forming three distinct clusters. These exhibited phylogenetic divergence from isolates obtained from marine fish of the Carangidae family (ST56 and ST115) from Japan and China (Figure 3).

The *L. garvieae* strains clustered into fourteen groups. The Brazilian isolates that were not from tilapia grouped independently or alongside other aquatic animal isolates worldwide within eight of these groups. The LG10-14 (ST105), LG66-22 (ST46) and 31MS (nST180) strains clustered with isolates obtained from disease outbreaks in Nile tilapia in Brazil (Figure 4).

The *L. petauri* strains clustered into nine distinct phylogenetic groups. The Brazilian isolates that were not from tilapia were distributed among five of these clusters, with the majority (82.1%) forming a single predominant cluster. The analysis revealed genetic divergence between these isolates and those obtained from disease outbreaks in rainbow trout in Europe (ST14), the United States (nST145) and Mexico (nST145). Notably, AM-LG02 and AM-LG03 strains clustered with ST47 and ST24 isolates, respectively, which originated from disease outbreaks in Brazilian tilapia farms (Figure 5).

## 4. Discussion

The present study investigated the population structure and genetic profile of a set of LCB strains obtained from different fish species in Brazil, using MLST as the genotyping method. Based on sequences deposited in the PubMLST database for *L. garvieae*—including the isolates reported in this study—80 STs were assigned to isolates derived from aquatic animals (Figure 2), which include isolates from both clinical disease cases, stool samples and fish meat products. Among these, 18 STs belong to *L. formosensis*, 29 to *L. petauri* and 33 to *L. garvieae* (Table 5), highlighting the genetic heterogeneity among these bacterial species.

When the MLST scheme was first developed by Ferrario et al. [22], all the isolates were believed to belong to *L. garvieae*, revealing two distinct genetic populations within the analyzed collection of strains. Subsequent studies, using strains from diverse sources (human, animal, food and environmental), identified a wide range of STs, which indicates the genetic heterogeneity of *L. garvieae* [23,24,31,32]. However, a study conducted in 2017 redefined the *L. garvieae* subgroup A as a new species, named *L. petauri*, and suggested the reassignment of previously characterized isolates [52]. Consequently, various studies have been conducted to improve the speciation within the genus *Lactococcus* [21,42,53]. It was only after 2023 that the first studies using the MLST approach to differentiate genetic profiles among LCB species were published [3,6,25,26], demonstrating high and comparable genetic diversity within each species, based on isolates from both human and animal sources [26]. During this same period, our research team constructed the *L. garvieae* MLST scheme in the PubMLST database. Since then, we have curated all the newly deposited sequences—including alleles, isolates and genomes—to ensure standardized nomenclature for major STs and CCs, integrating and consolidating data from LCB strains, and providing a comprehensive analysis of their genetic and epidemiological characteristics. Thus, by sequencing the seven housekeeping genes of our isolates and utilizing the PubMLST database (accessed on 5 August 2025), it was possible to compare the population structure and phylogenetic relationships of *Lactococcus* spp. strains obtained from fish in Brazil, with those of other countries.

Our results for this dataset demonstrate that the LCB isolates from the fish belong to 11 previously established STs. ST4 and ST122 were previously identified in animal-derived products, including fish meat, from China, Italy and Spain [22,26]. Notably, ST4, ST20, ST29 and ST105 have been associated with human diseases in China, Singapore, Spain and the United States [22,23,26,32]. Additional epidemiological findings include: ST6 reported in vegetable isolates from Italy; ST61 detected in water samples from Spain; ST25, ST35 and ST152 identified in human and swine fecal samples from China and Spain [22,23]. Among the previously reported STs, only ST6 and ST46 have been found in diseased fish, in the United States and Brazil, respectively [3,42]. The high values of the SDI show a considerable genetic divergence among the isolates evaluated, with *L. formosensis* and *L. garvieae* having greater ST richness and higher ST-level diversity in this study while *L. petauri* collection suggested a less diverse population.

It is important to mention that when evaluating the ancestry of the isolates through CC analysis, no cluster comprising three or more STs formed exclusively by isolates from aquatic animals was observed (Figure 2).

*L. garvieae* CC4, which groups ST4 (LG09-14 and LG63-21 strains) and ST122 (177 strain) identified in this study, appears to be associated with isolates from animal-derived products, particularly samples originating from the European continent [26]. Nonetheless, ST13, which also belongs to this CC, includes isolates from rainbow trout in Italy [22,42]. On the other hand, *L. garvieae* CC17 appears to harbor more STs (ST16, ST17, ST46, and ST139) associated with clinical manifestations of disease in fish [3,22,23,25]. This corroborates results from our study, as 31MS (nST180) and LG66-22 (ST46) strains, isolated from diseased *Pseudoplatystoma fasciatum* and *Phractocephalus hemioliopterus*, respectively, grouped within this CC. A previous study assessed the pathogenicity of the 31MS strain through experimental infection (10^7^ CFU/fish) in *Pseudoplatystoma* spp. [54]. During the 21-day monitoring period, 10.6% of the animals exhibited clinical signs of diseases; however, no mortality was observed. Conversely, the LG66-22 strain belongs to the same ST identified in disease outbreaks affecting Nile tilapia in Brazil in 2019 and 2021 [3]. Since the pathogenicity of this specific ST has not been evaluated, future laboratory controlled challenges comparing the susceptibility of Nile tilapia and *Phractocephalus hemioliopterus* is warranted to better understand the pathogenicity of this ST. Finally, *L. garvieae* CC62 includes isolates from fish in India and Spain (ST62 and ST157) [33,42], and is grouped with a strain from the current study, LG64-21 (nST176), which was obtained from an ornamental fish species. Although a few LCB isolates from ornamental fish were included in this study, the two *L. garvieae* strains possess different STs, showing no apparent consistent host- or region-associated pattern within the limits of this uneven isolate collection.

*L. formosensis* CC5 is also predominantly associated with isolates from animal-derived products, including fish meat from China (ST5 and ST113) [26]. In the current study, we did not identify any isolates belonging to this CC.

*L. petauri* CC24 comprises isolates associated with diseases in fish (Nile tilapia–ST24; catfish–nST142) and humans (ST24), as well from human (ST24) and swine (ST155) feces [3,23,32,42]. ST24 has been the predominant genetic profile among *L. petauri* isolates obtained from Nile tilapia in different types of commercial production and different geographic regions in Brazil between 2020 and 2022, and its pathogenicity and high virulence for this aquatic host were confirmed [3]. Interestingly, none of the isolates evaluated in this study shared this ST or belonged to CC24, suggesting that these isolates may have emerged from a distinct ancestor. On the other hand, *L. petauri* CC29 clustered isolates from diverse sources and was the largest CC identified in this study. CC29 clustered isolates from human feces [22,23], fish (cobia and European seabass) and prawn sashimi, such as ST10, ST128 and ST135 [26,32,42]. A total of 22 out of 28 *L. petauri* strains from our study belong to this CC, indicating that isolates of this bacterial species tend to have a more homogeneous genetic profile compared to the other two bacterial species investigated. Other studies utilizing different genotyping methods (DNA fingerprinting approaches) also revealed a more homogeneous population for *L. petauri* strains [21]. Finally, *L. petauri* CC97 contains isolates linked to diseases in fish (ST98 and nST145) and fish meat (ST137) [26,42]. Among these, nST145 has been associated with major mortality outbreaks in rainbow trout in the United States and Mexico between 2016 and 2020 [9,55]. No isolate from this study grouped within this CC.

Other genetic relationships were also identified via goeBURST analysis. For *L. petauri*: ST27 (human feces, Spain) and ST61 (LG03-18 strain); ST47 (Nile tilapia, Brazil) and nST181 (14MS strain); ST53 (bovine mastitis, Spain) and nST177 (AM-LG03 strain); ST34 (red tilapia, Singapore) and ST82 (human feces, China). For *L. garvieae*: nST165 (LG88-23, LG89-23 and PA-LG01 strains) and nST173 (CRBP138 and CRBP144) from Amazonian fish species; ST39 (tilapia, Singapore) and ST50 (bovine mastitis, Spain). And for *L. formosensis*: ST41 (carp, Singapore) and ST59 (fish, Spain); ST91 (bovine mastitis, China) and ST151 (barramundi, USA) [25,32,42]. Furthermore, our study observed that many isolates were singletons (lacking a common ancestor with other isolates), underscoring the significant genetic heterogeneity of these bacteria. Despite this, there are currently 181 STs deposited in the PubMLST database. In the future, with the addition of more isolates and allelic profiles, new population structure relationships among LCBs may be revealed.

Since the *Lactococcus* spp. isolates deposited in the PubMLST database originate from diverse sources, such as clinical samples from humans and animals, dairy and meat products, feces from healthy individuals, and even vegetables, uncertainties remain regarding the public health and food safety implications of these pathogens. Human infections have been reported following the handling or ingestion of raw fish and seafood [56,57,58], with the latter considered the most probable source of infection. For this reason, LCBs (especially *L. garvieae*) are considered potential zoonotic bacteria. However, the microbiological and molecular data available to support this hypothesis are scarce, as foodborne transmission from fish and seafood is usually inferred from clinical histories, with limited epidemiological evidence to confirm the transmission of the bacteria between food and humans [58]. Although our study and others in the scientific literature have demonstrated that isolates from different sources can share the same ST [22,23,26,31], it is currently not possible to determine whether a specific ST or CC is safe or potentially hazardous to humans.

Phylogenetic analysis of the concatenated housekeeping genes was used to reconstruct the evolutionary relationships among the strains of the tested bacterial species. As expected, the analysis enabled the grouping of strains within the same CC. However, it also revealed arrangements that represented double- or triple-locus variants. The analysis revealed that our *L. formosensis* strains are closely related to others obtained from largemouth bass (nST140 and nST141), barramundi (nST151), and rainbow trout (nST150) in the USA; from a fish with no designated species in Singapore (ST43); and from salmon (ST113) and flounder (ST5) sashimi in China [26,32,42]. The *L. garvieae* strains are related to those obtained from salmon (ST122) and prawn (ST119) sashimi (China), rainbow trout (ST62, India; ST63, Spain), European seabass (ST157 and ST158, Spain, [33]), unspecified fish species (ST6, USA), tilapia (ST39, Singapore; ST46, Brazil), yellowtail (ST16 and ST17, Japan), and so-iuy mullet (ST17, South Korea). This broad host range demonstrates a lack of host specificity and no clear phylogenetic distinction based on geographic origin. Conversely, the *L. petauri* strains demonstrated a more intriguing genetic relationship. Most of our isolates (those related to CC29) are genetically linked to other isolates obtained from tilapia (ST34, Singapore), cobia (ST10, Singapore), hybrid catfish (nST142, USA), European seabass (ST128, USA) and prawn sashimi (ST135, China) [26,32,42]. Isolates from clinical cases of piscine lactococcosis in trout were divided into two distinct phylogenetic clades: one associated with ST14, ST57 and nST146, identified primarily in European countries (with a single representative from the USA and Canada), and another clade associated with nST145, which, as previously mentioned, is linked to recent and impactful outbreaks in North America. This division presents a strong geographical signal of diversification among trout isolates. Our isolates are not phylogenetically related to these clades, indicating they evolved from different ancestors. In contrast, two of our *L. petauri* strains (AM-LG02 and AM-LG03), despite some phylogenetic distance, share a common ancestor with isolates associated with disease outbreaks in Nile tilapia in Brazil. Both isolates were obtained from the intestine of *Colossoma macropomum*. In an experimental infection study in this same aquatic host, the AM-LG02 strain did not cause any macroscopic or microscopic alterations in the challenged animals [59]. Therefore, future studies should use this and other LCB isolates in challenge experiments with tilapia to verify their pathogenic potential for this species.

## 5. Conclusions

This study provides new insights into the genetic diversity of Brazilian *Lactococcus* spp. strains isolated from different fish species, using an MLST approach. The analysis revealed that LCB isolates constitute a genetically diverse population based on their STs. Specifically, *L. garvieae* and *L. formosensis* exhibited greater heterogeneity compared to *L. petauri*, for which the majority of isolates belonged to a single clonal complex (CC29). MLST and phylogenetic analysis demonstrated genetic relatedness between the *L. garvieae* and *L. formosensis* isolates from this study and those from other aquatic animal species deposited in the PubMLST database. Regarding the *L. petauri* strains, all the STs identified in this study were unrelated to the MLST lineages responsible for outbreaks in Brazilian Nile tilapia and North American rainbow trout. This suggests that piscine *L. petauri* populations in the Americas evolved from distinct ancestral origins. However, phylogenetic analysis and MLST data showed that, although they belong to different CC, two isolates from *Colossoma macropomum* are genetically closely related to isolates from Nile tilapia in Brazil. Therefore, future studies, particularly those employing a whole-genome sequencing approach, are necessary to better elucidate the ancestral relationship between these strains. The results obtained herein provide a better understanding of the genetic diversity of *Lactococcus* spp. populations in fish from Brazil. Additionally, these findings will contribute to future molecular epidemiology studies, facilitating the selection of candidate strains for whole-genome sequencing, as well as the assessment of the pathogenesis and evolutionary aspects of LCBs using in vivo (experimental infection) or in vitro (transcriptomics and proteomics) models.

## 6. Limitations

Quantitatively, the Brazilian non-tilapia collection analyzed here represented a substantial fraction of the aquatic-animal ST diversity currently available in PubMLST. Among aquatic animal-derived STs, this study identified 6/18 (33.3%) *L. formosensis* STs, 14/33 (42.4%) *L. garvieae* STs and 9/29 (31.0%) *L. petauri* STs. Overall, the 29 STs detected in this study accounted to 29/80 (36.3%) of all aquatic animal-derived STs in the dataset analyzed, and 18/29 (62.1%) were novel. Compared with previous Brazilian MLST data for Nile tilapia, which reported only ST24 and ST47 for *L. petauri* and ST46 for *L. garvieae*, the present non-tilapia collection substantially expands the known Brazilian ST spectrum. However, this study was designed as a retrospective descriptive MLST survey of Brazilian *Lactococcus* spp. isolates from non-tilapia fish, rather than as a population-based epidemiological study. Therefore, ST and CC frequencies should be interpreted as patterns observed within this isolate collection, rather than as estimates of genotype prevalence in Brazilian aquaculture. Further, because host species, tissue, year and production origin were unevenly distributed and many categories were represented by one or two isolates, formal association tests for these variables were considered underpowered and were not used to support epidemiological conclusions.

## Figures and Tables

**Table 1:** Metadata of the 55 *Lactococcus* spp. analyzed in this study.

Isolate	Species	Host	Origin	Tissue	Year	State	Collection Site	Culture Collection	Reference
167/23-02	*L. formosensis*	*Arapaima gigas*	Farmed	Brain	2023	BA	1	AQUAVET	[21]
167/23-06	*L. formosensis*	*Arapaima gigas*	Farmed	Brain	2023	BA	1	AQUAVET	[21]
167/23-09	*L. formosensis*	*Arapaima gigas*	Farmed	Kidney	2023	BA	1	AQUAVET	[39]
AM-LG05	*L. formosensis*	*Colossoma macropomum*	Farmed	Intestine	2022	AM	2	LAMAO	[21]
49/21-29	*L. formosensis*	*Pangasianodon hypophthalmus*	Farmed	Brain	2021	SP	3	AQUAVET	This study
52MS	*L. formosensis*	*Pseudoplatystoma fasciatum*	Farmed	Brain	2012	MS	4	LAPOA	[35]
LG91-23	*L. formosensis*	*Pseudoplatystoma* sp.	Farmed	Brain	2023	MG	5	AQUAVET	[21]
CRBP53	*L. garvieae*	*Arapaima gigas*	Farmed	Intestine	2023	AM	6	LAMAO	[21]
CRBP54	*L. garvieae*	*Arapaima gigas*	Farmed	Intestine	2023	AM	6	LAMAO	[21]
CRBP138	*L. garvieae*	*Arapaima gigas*	Farmed	Intestine	2023	AM	7	LAMAO	[21]
CRBP144	*L. garvieae*	*Arapaima gigas*	Farmed	Intestine	2023	AM	7	LAMAO	[21]
PA-LG01	*L. garvieae*	*Arapaima gigas*	Farmed	Brain	2018	PA	8	LAMAO	[36]
LG88-23	*L. garvieae*	*Brycon amazonicus*	Farmed	Brain	2023	MG	5	AQUAVET	[21]
LG89-23	*L. garvieae*	*Brycon amazonicus*	Farmed	Kidney	2023	MG	5	AQUAVET	[21]
LG116-23	*L. garvieae*	*Cichla* sp.	Wild	Brain	2023	MG	9	AQUAVET	[39]
LG63-21	*L. garvieae*	*Hoplias macrophtalmus*	Farmed	Kidney	2021	MG	10	AQUAVET	[21]
LG114-23	*L. garvieae*	*Hoplias malabaricus*	Wild	Brain	2023	AM	11	AQUAVET	[39]
LG10-14	*L. garvieae*	*Lophiosilurus alexandri*	Farmed	Brain	2014	MG	12	AQUAVET	[37]
49/21-11	*L. garvieae*	*Pangasianodon hypophthalmus*	Farmed	Kidney	2021	SP	3	AQUAVET	This study
LG66-22	*L. garvieae*	*Phractocephalus hemioliopterus*	Farmed	Kidney	2022	MG	13	AQUAVET	[21]
LG09-14	*L. garvieae*	*Pseudoplatystoma corruscans*	Farmed	Kidney	2014	SP	14	AQUAVET	[37]
LG23-16	*L. garvieae*	*Pseudoplatystoma corruscans*	Farmed	Brain	2016	SP	15	AQUAVET	[38]
177	*L. garvieae*	*Pseudoplatystoma fasciatum*	Farmed	Brain	2012	MS	16	IP	[16]
31MS	*L. garvieae*	*Pseudoplatystoma fasciatum*	Farmed	Kidney	2012	MS	4	LAPOA	[35]
LG119-24	*L. garvieae*	*Pseudoplatystoma* sp.	Farmed	Brain	2024	MG	17	AQUAVET	[39]
LG115-23	*L. garvieae*	*Trichogaster lalius*	Farmed	Kidney	2023	MG	18	AQUAVET	This study
LG64-21	*L. garvieae*	*Xiphophorus maculatus*	Farmed	Kidney	2021	MG	19	AQUAVET	This study
167/23-03	*L. petauri*	*Arapaima gigas*	Farmed	Kidney	2023	BA	1	AQUAVET	[39]
167/23-04	*L. petauri*	*Arapaima gigas*	Farmed	Kidney	2023	BA	1	AQUAVET	[39]
167/23-05	*L. petauri*	*Arapaima gigas*	Farmed	Kidney	2023	BA	1	AQUAVET	[39]
167/23-07	*L. petauri*	*Arapaima gigas*	Farmed	Kidney	2023	BA	1	AQUAVET	[39]
167/23-08	*L. petauri*	*Arapaima gigas*	Farmed	Kidney	2023	BA	1	AQUAVET	[39]
167/23-10	*L. petauri*	*Arapaima gigas*	Farmed	Spleen	2023	BA	1	AQUAVET	[39]
CRBP89	*L. petauri*	*Arapaima gigas*	Farmed	Intestine	2023	AM	20	LAMAO	[21]
CRBP98	*L. petauri*	*Arapaima gigas*	Farmed	Intestine	2023	AM	20	LAMAO	[21]
CRBP146	*L. petauri*	*Arapaima gigas*	Farmed	Intestine	2023	AM	20	LAMAO	[21]
AM-LG07	*L. petauri*	*Brycon amazonicus*	Farmed	Brain	2022	AM	21	LAMAO	[21]
AM-LG08	*L. petauri*	*Brycon amazonicus*	Farmed	Brain	2022	AM	21	LAMAO	[21]
LG120-24	*L. petauri*	*Carassius auratus*	Farmed	Kidney	2024	MG	22	AQUAVET	This study
LG121-24	*L. petauri*	*Carassius auratus*	Farmed	Kidney	2024	MG	22	AQUAVET	This study
AM-LG02	*L. petauri*	*Colossoma macropomum*	Farmed	Intestine	2020	AM	23	LAMAO	[21]
AM-LG03	*L. petauri*	*Colossoma macropomum*	Farmed	Intestine	2022	AM	2	LAMAO	[21]
49/21-21	*L. petauri*	*Pangasianodon hypophthalmus*	Farmed	Kidney	2021	SP	3	AQUAVET	This study
LG03-18	*L. petauri*	*Pseudoplatystoma corruscans*	Farmed	Brain	2018	MG	24	AQUAVET	[21]
14MS	*L. petauri*	*Pseudoplatystoma fasciatum*	Farmed	Kidney	2012	MS	4	LAPOA	[35]
176	*L. petauri*	*Pseudoplatystoma fasciatum*	Farmed	Brain	2012	MS	16	IP	[16]
86	*L. petauri*	*Pseudoplatystoma* sp.	Farmed	Brain	2012	MS	16	IP	[16]
89/2	*L. petauri*	*Pseudoplatystoma* sp.	Farmed	Brain	2012	MS	16	IP	[16]
93	*L. petauri*	*Pseudoplatystoma* sp.	Farmed	Brain	2012	MS	16	IP	[16]
LG86-23	*L. petauri*	*Pseudoplatystoma* sp.	Farmed	Kidney	2023	MG	5	AQUAVET	[21]
LG94-23	*L. petauri*	*Pseudoplatystoma* sp.	Farmed	Brain	2023	MG	5	AQUAVET	[21]
LG104-23	*L. petauri*	*Pseudoplatystoma* sp.	Farmed	Brain	2023	MG	5	AQUAVET	[21]
LG106-23	*L. petauri*	*Pseudoplatystoma* sp.	Farmed	Kidney	2023	MG	5	AQUAVET	[21]
LG117-23	*L. petauri*	*Pseudoplatystoma* sp.	Farmed	Kidney	2023	MG	25	AQUAVET	[39]
AM-LG06	*L. petauri*	*Pterophyllum scalare*	Farmed	Liver	2022	AM	26	LAMAO	This study

AM: Amazonas; BA: Bahia; MS: Mato Grosso do Sul; MG: Minas Gerais; PA: Pará; SP: São Paulo.

**Table 2:** Oligonucleotide primers used in the MLST assay for *Lactococcus* spp. strains and polymorphism observed for each gene.

Gene	Primer Pairs (5′-3′)	Annealing Temperature (°C)	Size (bp)	N° of Alleles	N° of Polymorphic Sites	Haplotypic Diversity	Nucleotide Diversity
*als*	F: ATTCGGCTCAGACTTAGTTG R: TTCAGCTGCTTCAACATCAA	58	811	100	167	1.000 ± 0.0014	0.03195
*atpA*	F: TAYRTYGGKGAYGGDATYGC R: CCRCGRTTHARYTTHGCYTG	56	803	69	236	1.000 ± 0.002	0.03465
*tuf*	F: ATATGCGGCCGCCATYGGHCACGTBGACCA R: AAAATATGCGGCCGCTCNCCNGGCATNACCAT	56	809	59	170	1.000 ± 0.003	0.01730
*gapC*	F: AAGTTGGTATTAACGGTTTCG R: AAGTGTACGAACGAGGTTAG	56	821	41	51	1.000 ± 0.005	0.00569
*gyrB*	F: CATGCTGGTGGTAAATTTGG R: GTCATCCATTTCTCCTAAACC	58	827	75	204	1.000 ± 0.002	0.05683
*rpoC*	F: TTGGTCCACAAAAGGACTGG R: TCACGTCCTTTTGCTTCCAT	58	830	66	117	1.000 ± 0.003	0.02684
*galP*	F: TGGGGAAAATTTAAACCTTGG R: ATCATCAGAACGGCTGGAAG	58	812	83	213	1.000 ± 0.002	0.05746

**Table 3:** Characteristics and allelic profiles of the Brazilian *Lactococcus* spp. isolates analyzed in this study.

Isolate	Species	Host	MLST								
			Allele							ST	CC
			*als*	*atpA*	*tuf*	*gapC*	*gyrB*	*rpoC*	*galP*		
167/23-02	*L. formosensis*	*Arapaima gigas*	22	62	18	3	20	4	78	n168	Singleton
167/23-06	*L. formosensis*	*Arapaima gigas*	15	10	14	9	13	15	15	20	Singleton
167/23-09	*L. formosensis*	*Arapaima gigas*	22	62	18	3	20	4	78	n168	Singleton
49/21-29	*L. formosensis*	*Pangasianodon hypophthalmus*	100	4	18	3	20	4	79	n174	Singleton
52MS	*L. formosensis*	*Pseudoplatystoma fasciatum*	91	60	14	9	20	33	81	n179	Singleton
AM-LG05	*L. formosensis*	*Colossoma macropomum*	90	35	14	9	20	38	81	n178	Singleton
LG91-23	*L. formosensis*	*Pseudoplatystoma* sp.	92	4	50	3	20	4	73	n166	Singleton
177	*L. garvieae*	*Pseudoplatystoma fasciatum*	3	3	4	2	59	3	3	122	CC4
31MS	*L. garvieae*	*Pseudoplatystoma fasciatum*	12	8	54	7	27	13	12	n180	CC17
49/21-11	*L. garvieae*	*Pangasianodon hypophthalmus*	5	5	6	2	5	5	5	6	Singleton
CRBP53	*L. garvieae*	*Arapaima gigas*	93	61	51	15	72	61	74	n167	Singleton
CRBP54	*L. garvieae*	*Arapaima gigas*	93	61	51	15	72	61	74	n167	Singleton
CRBP138	*L. garvieae*	*Arapaima gigas*	34	59	27	15	28	19	29	n173	-
CRBP144	*L. garvieae*	*Arapaima gigas*	34	59	27	15	28	19	29	n173	-
LG09-14	*L. garvieae*	*Pseudoplatystoma corruscans*	3	3	4	2	3	3	3	4	CC4
LG10-14	*L. garvieae*	*Lophiosilurus alexandri*	60	8	6	7	10	45	48	105	Singleton
LG23-16	*L. garvieae*	*Pseudoplatystoma corruscans*	88	22	46	25	71	20	71	n164	Singleton
LG63-21	*L. garvieae*	*Hoplias macrophtalmus*	3	3	4	2	3	3	3	4	CC4
LG64-21	*L. garvieae*	*Xiphophorus maculatus*	87	24	27	15	28	27	29	n176	nCC62
LG66-22	*L. garvieae*	*Phractocephalus hemioliopterus*	12	8	6	7	27	13	12	46	CC17
LG88-23	*L. garvieae*	*Brycon amazonicus*	34	59	27	15	28	19	72	n165	-
LG89-23	*L. garvieae*	*Brycon amazonicus*	34	59	27	15	28	19	72	n165	-
LG114-23	*L. garvieae*	*Hoplias malabaricus*	34	59	27	15	28	19	72	n165	-
LG115-23	*L. garvieae*	*Trichogaster lalius*	21	13	1	2	73	16	75	n169	Singleton
LG116-23	*L. garvieae*	*Cichla* sp.	94	3	52	2	73	62	76	n170	Singleton
LG119-24	*L. garvieae*	*Pseudoplatystoma* sp.	95	69	6	2	5	63	77	n171	Singleton
PA-LG01	*L. garvieae*	*Arapaima gigas*	34	59	27	15	28	19	72	n165	-
86	*L. petauri*	*Pseudoplatystoma* sp.	9	7	3	2	37	9	9	152	nCC29
93	*L. petauri*	*Pseudoplatystoma* sp.	9	7	3	2	37	9	9	152	nCC29
176	*L. petauri*	*Pseudoplatystoma fasciatum*	9	7	3	4	16	9	17	25	Singleton
14MS	*L. petauri*	*Pseudoplatystoma fasciatum*	32	21	7	2	7	11	6	n181	-
167/23-03	*L. petauri*	*Arapaima gigas*	94	6	7	2	7	11	82	n172	Singleton
167/23-04	*L. petauri*	*Arapaima gigas*	9	7	3	4	18	9	9	29	nCC29
167/23-05	*L. petauri*	*Arapaima gigas*	9	7	3	4	18	9	9	29	nCC29
167/23-07	*L. petauri*	*Arapaima gigas*	9	7	3	4	18	9	9	29	nCC29
167/23-08	*L. petauri*	*Arapaima gigas*	9	7	3	4	18	9	9	29	nCC29
167/23-10	*L. petauri*	*Arapaima gigas*	9	7	3	4	18	9	9	29	nCC29
49/21-21	*L. petauri*	*Pangasianodon hypophthalmus*	9	7	3	4	18	9	9	29	nCC29
89/2	*L. petauri*	*Pseudoplatystoma* sp.	9	7	3	2	37	9	9	152	nCC29
AM-LG02	*L. petauri*	*Colossoma macropomum*	61	6	7	35	7	11	8	n175	Singleton
AM-LG03	*L. petauri*	*Colossoma macropomum*	89	20	26	2	24	25	6	n177	-
AM-LG06	*L. petauri*	*Pterophyllum scalare*	9	7	3	2	7	9	9	35	nCC29
AM-LG07	*L. petauri*	*Brycon amazonicus*	9	7	3	2	7	9	9	35	nCC29
AM-LG08	*L. petauri*	*Brycon amazonicus*	9	7	3	2	7	9	9	35	nCC29
CRBP89	*L. petauri*	*Arapaima gigas*	9	7	3	2	7	9	9	35	nCC29
CRBP98	*L. petauri*	*Arapaima gigas*	9	7	3	2	7	9	9	35	nCC29
CRBP146	*L. petauri*	*Arapaima gigas*	9	7	3	2	7	9	9	35	nCC29
LG03-18	*L. petauri*	*Pseudoplatystoma corruscans*	33	6	10	2	7	11	8	61	-
LG86-23	*L. petauri*	*Pseudoplatystoma* sp.	9	7	3	4	18	9	9	29	nCC29
LG94-23	*L. petauri*	*Pseudoplatystoma* sp.	9	7	3	4	18	9	9	29	nCC29
LG104-23	*L. petauri*	*Pseudoplatystoma* sp.	9	7	3	4	18	9	9	29	nCC29
LG106-23	*L. petauri*	*Pseudoplatystoma* sp.	9	7	3	4	18	9	9	29	nCC29
LG117-23	*L. petauri*	*Pseudoplatystoma* sp.	9	7	3	4	18	9	9	29	nCC29
LG120-24	*L. petauri*	*Carassius auratus*	9	7	3	4	18	9	9	29	nCC29
LG121-24	*L. petauri*	*Carassius auratus*	9	7	3	4	18	9	9	29	nCC29

**Table 4:** Sequence type diversity and clonal-complex summary of isolates.

Group/Species	Number of Isolates	#STs	Number of New STs	Predominant ST	ST with Only 1 Isolate	Simpson, IC 95%	CC Summary
Total	55	29	18/29, 62.1%	ST29, 13/55, 23.6%	21	0.929, 0.883–0.975	multiple CCs and many singletons
*L. formosensis*	7	6	5/6, 83.3%	nST168, 2/7, 28.6%	5	0.952, 0.857–1.000	all singletons
*L. garvieae*	20	14	9/14, 64.3%	nST165, 4/20, 20.0%	10	0.953, 0.903–1.000	CC4: 3/20; CC17: 2/20; nCC62: 1/20; 14 with no defined CC
*L. petauri*	28	9	4/9, 44.4%	ST29, 13/28, 46.4%	6	0.746, 0.612–0.880	nCC29: 22/28, 78.6%

**Table 5:** Number of aquatic animal-derived sequence types identified in this and previous studies, categorized by bacterial species.

Bacterial Species	ST in Aquatic Animals/ST Total ^a^	STs Identified in This Study	STs Identified in Other Studies
*L. formosensis*	18/39	ST20, nST166, nST168, nST174. nST178, nST179	ST5, ST41, ST43, ST56, ST59, ST113, ST114, ST115, nST140, nST141, nST150, nST151
*L. garvieae*	33/55	ST4, ST6, ST46, ST105, ST122, nST164, nST165, nST167, nST169, nST170, nST171, nST173, nST176, nST180	ST1, ST13, ST16, ST17, ST39, ST62, ST63, ST95, ST109, ST119, ST120, ST121, ST123, ST124, ST139, nST144, nST147, ST157, ST158
*L. petauri*	29/85	ST25, ST29, ST35, ST61, ST152, nST172, nST175, nST177, nST181	ST10, ST14, ST15, ST24, ST34, ST47, ST57, ST98, ST128, ST132, ST133, ST134, ST135, ST136, ST137, ST138, nST142, nST145, nST146, nST149
*Lactococcus* ssp. ^b^	0/2	-	-
Total	80/181	29/181	51/181

^a^ Proportion of sequence types identified from aquatic animal isolates relative to the total number of STs deposited in PubMLST; ^b^ Strains currently classified as *Lactococcus garvieae* in PubMLST database but shown by genomic analysis to represent a distinct, yet taxonomically uncharacterized *Lactococcus* species [42].

## Data Availability

The data that support the findings of this study are available on request from the corresponding author.

## Supplementary Materials

The following supporting information can be downloaded at: https://www.mdpi.com/article/10.3390/microorganisms14051131/s1, Table S1: *Lactococcus* ssp. strains isolated from aquatic animals with publicly available genome sequences in GenBank databases included in this study; Table S2: *Lactococcus* spp. strains isolated from diverse animal aquatic species with previously reported alleles and sequence type included in this study to phylogenetic analyses.
